# Insulin-Like Growth Factor 1 Receptor (IGF-1R) as a Target of MiR-497 and Plasma IGF-1R Levels Associated with TNM Stage of Pancreatic Cancer

**DOI:** 10.1371/journal.pone.0092847

**Published:** 2014-03-25

**Authors:** Jian-Wei Xu, Tian-Xiao Wang, Lei You, Lian-Fang Zheng, Hong Shu, Tai-Ping Zhang, Yu-Pei Zhao

**Affiliations:** 1 Department of General Surgery, Peking Union Medical College Hospital, Chinese Academy of Medical Sciences and Peking Union Medical College, Beijing, China; 2 Department of Head and Neck Surgery, Beijing Cancer Hospital and Institute, Peking University Cancer Hospital, Beijing, China; 3 Department of Nuclear Medicine, Peking Union Medical College Hospital, Chinese Academy of Medical Sciences and Peking Union Medical College, Beijing, China; Thomas Jefferson University, United States of America

## Abstract

The expression levels and regulatory roles of miR-497 in pancreatic cancer are unclear. The clinical value of plasma insulin-like growth factor 1 receptor (IGF-1R) in pancreatic cancers has not been investigated. In the present study, we demonstrated that miR-497 was significantly downregulated in pancreatic cancer tissues. Upregulation of miR-497 in BxPC-3 and AsPC-1 pancreatic cancer cell lines inhibited proliferation, enhanced apoptosis, re-sensitized cells to gemcitabine and suppressed IGF-1R and p-AKT expression through direct downregulation of IGF-1R protein expression. Opposite effects were observed after downregulation of miR-497. Plasma IGF-1R levels in patients with pancreatic cancer increased significantly, compared with that in patients with chronic pancreatitis, other pancreatic tumors and pancreatic neuroendocrine tumors (*P = 0.006*, *P = 0.018* and *P = 0.004*, respectively), and displayed potential values for distinguishing pancreatic lesions. However, the levels in pancreatic cancer patients were comparable to that in healthy volunteers (*P = 0.095*). The tumor locations and TNM stage were associated with plasma IGF-1R levels (*P = 0.013* and *P = 0.01*, respectively). There was no significant difference of overall survival between high and low IGF-1R expression groups. In conclusion, we demonstrated that miR-497 attenuated the malignancy of pancreatic cancer cells and promoted sensitivity of cells to gemcitabine by directly downregulation of IGF-1R expression. Plasma IGF-1R displayed a potential value for distinguishing pancreatic lesions and could be a new biomarker for guiding TNM stage of pancreatic cancer.

## Introduction

Pancreatic ductal adenocarcinoma (PDAC) is an aggressive and devastating disease. PDAC has an extremely poor prognosis with a 5-year survival rate lower than 5% [1]. Although the mechanisms of pancreatic carcinogenesis, the new markers for early detection, and the new therapeutic strategies have been widely investigated [2], [3], [4], the overall survival has not been improved in the past 80 years [5]. The molecular mechanisms of the progression of PDAC, including proliferation, apoptosis, drug resistance, remain unclear.

MiRNAs are short non-coding RNAs, which can inhibit translation of messenger RNA (mRNA) into protein by binding to the 3′-untranslated region (3′-UTR). MiRNAs can act as oncogenes or tumor suppressors in the regulation of carcinogenesis, metastatic capacity and drug resistance [6]. Downregulation of miR-497 has been observed in breast, colorectal and cervical cancers [7], [8], [9]. However, there is no report about miR-497 expression levels in pancreatic cancer at present. Insulin-like growth factor 1 receptor (IGF-1R) was identified as a target of miR-497. Downregulation of miR-497 contributes to the malignancy of colorectal cancer and cervical cancer by upregulating IGF-1R [8], [9]. However, the regulatory roles and mechanisms of miR-497 in pancreatic cancer are still unclear.

IGF-1R is a tyrosine kinase receptor, which involves in the regulation of proliferation, apoptosis, differentiation and malignant transformation of cancer cells [10]. Upregulation of IGF-1R in human PDAC tissues has been reported [11] and associates with higher tumor grade and poor survival [12]. Nevertheless, the plasma IGF-1R levels in pancreatic cancer patients were not detected in previous studies. The clinical values of plasma IGF-1R in pancreatic cancer are unknown.

In the present study, we found that miR-497 was significantly downregulated in pancreatic cancer tissues. Upregulation of miR-497 inhibited proliferation, enhanced apoptosis and promoted sensitivity to gemcitabine through directly downregulating IGF-1R expression in PDAC cancer cells *in vitro*. We also showed that the levels of plasma IGF-1R in pancreatic cancer patients were comparable to that in healthy volunteers, but higher than that in patients with chronic pancreatitis, other pancreatic tumors and pancreatic neuroendocrine tumors, and displayed values for distinguishing pancreatic lesions. The tumor locations and TNM stage were associated with plasma IGF-1R levels. There was no significant difference of overall survival time between high and low IGF-1R expression groups.

## Methods

### Ethics statement

Studies were performed in accordance with ethics approval from the Institutional Review Boards of Peking Union Medical College Hospital. Written informed consent was obtained from all subjects.

### Detecting the expression of miR-497 by in situ hybridization (ISH)

10 formalin-fixed, paraffin-embedded pancreatic cancer specimens and matched tumor-adjacent tissues were obtained and made into tissue microarrays. The expression levels of miR-497 in tissues were detected using miRCURY LNA detection probe for miR-497 (Exiqon, Vedbaek, Denmark, product number: 38256-15). ISH was performed as following description. Briefly, slides were incubated at 37°C for 30 min, deparaffinized in xylene, and rehydrated with graded alcohol washes. Then slides were placed into 4% paraformaldehyde for 20 min in a fume hood, and then washed with phosphate buffered saline (PBS) three times. The slides were then treated with 15 μg/ml proteinase K for 15 min at room temperature. Thereafter, washed the slides with PBS and fixed them in 4% paraformaldehyde for 15 min after that. After rinsing, slides were pre-hybridized with hybridization buffer for 1 hour at room 50°C and then hybridized overnight at 4°C in the hybridization buffer containing probe. Stringent washes were carried out at 50°C for 20 min, and then the slides were incubated in a blocking solution for 1 hour at room temperature. Subsequently, slides were incubated in blocking solution with alkaline phosphatase conjugated anti-DIG Fab fragment overnight at 4°C. The colorimetric detection reaction was performed using NBT/ BCIP kit (ThermoFisher Scientific) according to the manufacturer’s protocol. The ISH results were recorded as the percentage of positive cells.

### Cell culture and reagents

BxPC-3 and AsPC-1 PDAC cell lines were kindly provided by Professor Helmut Freiss (Heidelberg University, Germany), who obtained from the American Tissue Type Culture Collection (ATCC, Rockville, MD) [13], [14], [15]. PDAC cells were cultured in a humidified incubator with 5% CO2 at 37°C in RPMI-1640 medium supplemented with 10% fetal bovine plasma (FBS, Hyclone). The primary antibodies were purchased from Cell Signaling Technology, including IGF-I Receptor β (D23H3) XP Rabbit mAb ( #9750), β-actin (13E5) Rabbit mAb (#4970), Phospho-Akt (Ser473) (D9E) XPRabbit mAb (#4060), Akt (pan) (11E7) Rabbit mAb (#4685), Caspase-3 Antibody ( #9662) and PARP Antibody ( #9542).

### MiRNA transfection

MiR-497 mimics (5′- CAGCAGCACACUGUGGUUUGU-3′, 5′-AAACCACAGUGUGCUGCUGUU-3′), mimics control (5′-UUCUCCGAACGUGUCACGUTT-3′, 5′-ACGUGACACGUUCGGAGAATT-3′), miR-497 inhibitor (5′-ACAAACCACAGUGUGCUGCUG-3′) and inhibitor control (5′-CAGUACUUUUGUGUAGUACAA-3′) were synthesized by Genepharma (Shanghai, China). MiRNAs at 50–100 nM were transfected using Lipofectamine 2000 transfection reagent (Invitrogen, Carlsbad, CA) according to the manufacturer’s protocol.

### Cellular RNA extraction and quantitative RT-PCR (qRT-PCR) assay

Cells were transfected in 6-well plates. After 48 hours of transfection, total RNA was extracted using TRIzol (Invitrogen, Carlsbad, CA) according to the manufacturer’s protocol. For IGF-1R quantitative assay, total RNA was reverse transcribed using the reverse transcription kit (Promega, Madison, WI) according to the manufacturer’s instructions. Real-time PCR was performed using the SYBR Green Master Mix (Takara, Japan). GAPDH were served as the endogenous control.

IGF-1R Forward primer: 5′-TCTGGCTTGATTGGTCTGGC-3′

Reverse primer: 5′-AACCATTGGCTGTGCAGTCA-3′

GAPDH Forward primer: 5′-CGGAGTCAACGGATTTGGTCGTAT-3′,

Reverse primer: 5′-AGCCTTCTCCATGGTGGTGAAGAC-3′

For miR-497 quantitative assay, TaqMan miRNA assay were used according to the manufacturer’s protocol (Applied Biosystems). U6 was used as an endogenous control. Fold changes were calculated using the 2^−ΔΔCT^ method.

### Proliferation analysis

*In vitro* proliferation was analyzed using a cell count kit (CCK-8). BxPC-3 cells and AsPC-1 cells were transfected in 6-well plates (5×10^5^cells/well). After 24 hours, cells were trypsinized and reseeded in 96-well plates (1000 cells/well). 10 μl/well CCK-8 reagent was added at 0, 24, 48, 72 hours, respectively, and incubated for 2.5 hours at 37°C. Optical density (OD) was measured at 450 nm and 630 nm by a microplate reader (Wellscan MK3, Thermo/Labsystems, Finland).

### Chemosensitivity analysis

BxPC-3 and AsPC-1 cells were transfected for 24 hours, plated in 96-well plates (4000 cells/well), and treated with serially diluted gemcitabine (Eli Lilly and Company) in triplicates. After 48 hours incubation, 10 μl/well CCK-8 reagent was added and incubated for 2.5 hours at 37°C. Optical density (OD) was measured at 450 nm and 630 nm using a microplate reader.

### Western blotting

After 48 hours of transfection in 6-well plates, cells were digested with trypsin solution and lysed with RIPA buffer (Applygen, Beijing). Total proteins were separated by sodium dodecyl sulfate polyacrylamide gel electrophoresis (SDS-PAGE) and transferred to a polyvinylidene difluoride (PVDF) membrane (Millipore, Billerica, MA). After blocking with 5% non-fat dry milk at room temperature for 1 hour, the membranes were incubated overnight at 4°C with primary antibodies. The membranes were then washed and incubated with a horseradish peroxidase-conjugated secondary antibody (Applygen, Beijing) at room temperature for 1 hour. Protein bands were visualized with echochemiluminescence (ECL) detection system, and the expression levels of these proteins were evaluated using Image-Pro Plus 6.0 software (Media Cybernetics, USA).

### Dual-luciferase reporter assay

The pmiR-RB-Report-IGF-1R-3′-UTR vectors containing wild type or mutated target sequence were constructed by RiboBio Co., Ltd. (Guangzhou, China). BxPC-3 cells were plated into 12 well plates (1×10^5^ cells/well) and co-transfected with miR-497 mimics and vectors expressing mutated target sequence, or co-transfected with mimics and vectors expressing wild type target sequence using lipofectamine 2000. After transfection for 48 hours, luciferase activity was measured using the Dual-Luciferase Reporter Assay System (Promega) according to the manufacturer’s protocol.

### Patients and plasma IGF-1R expression

Plasma samples were collected from 42 pancreatic cancer patients. Plasma samples from patients with other pancreatic tumors (29 patients, including serous cystadenoma (7 cases), mucinous cystadenoma (8 cases), solid pseudopapillary tumor (10 cases) and intra-ductal papillary mucinous neoplasm (4 cases)), chronic pancreatitis (CP, 19 patients), pancreatic neuroendocrine tumor (PNET, 19 patients) and healthy volunteers (30 cases) were collected as controls. Pancreatic cancer, PNET and other pancreatic tumors were diagnosed through pathological examination. CP was diagnosed according to the clinical diagnostic criteria. Blood samples were centrifuged at 3000 revolutions per minute (rpm) for 10 minutes. Plasma was collected and stored at –80°C before use. The plasma IGF-1R levels were detected using human IGF-1R ELISA Kit (Catalog number: CSB-E13766h, CUSABIO, China) following the manufactureŕs protocol.

### Statistical analysis

SPSS v.13.0 software (SPSS, Inc, Chicago, IL) was used for statistical analyses. Continuous data were presented as mean ± standard deviation (SD) and compared by analysis of variance (ANOVA), student’s *t* test or the Mann–Whitney *U* test. Categorical data were presented as percentage and compared using a Pearson χ^2^ test or Fisher exact test when cell counts were <5. The Kaplan-Meier method was used for survival analysis using the log-rank test. Statistical significance was defined as *P < 0.05*.

## Results

### MiR-497 was downregulated in pancreatic cancer tissues

**Figure 1:**
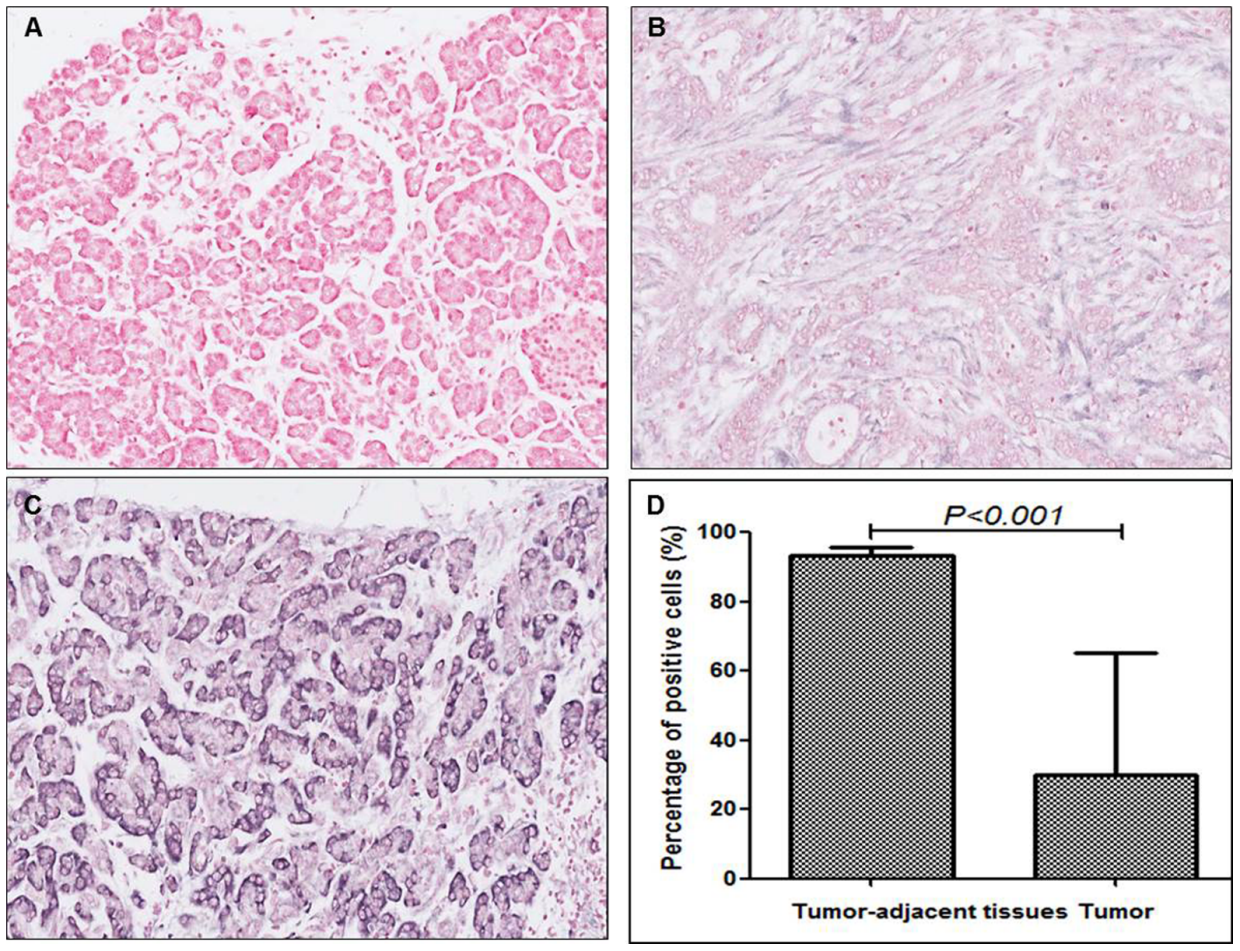
The expression level of miR-497 in pancreatic tissues. MiR-497 levels in pancreatic tissues were detected using ISH (200×). (**A**) Negative control. (**B**) ISH for miR-497 in pancreatic cancer tissue. (**C**) ISH for miR-497 in tumor-adjacent tissue. (**D**) The average percentage of positive cells in pancreatic cancer tissues was 30.0%±35.4%, which was significantly lower than that in tumor-adjacent tissues (93.5.0%±2.4%). The data were shown as mean±SD.

MiR-497 levels in 10 pancreatic cancer specimens and matched tumor-adjacent tissues were detected using ISH. The average percentage of positive cells in pancreatic cancer tissues was 30.0%±35.4%, which was significantly lower than that in tumor-adjacent tissues (93.5.0%±2.4%) (*P = 0.000*).(**Figure 1**)

### MiR-497 inhibited proliferation

**Figure 2:**
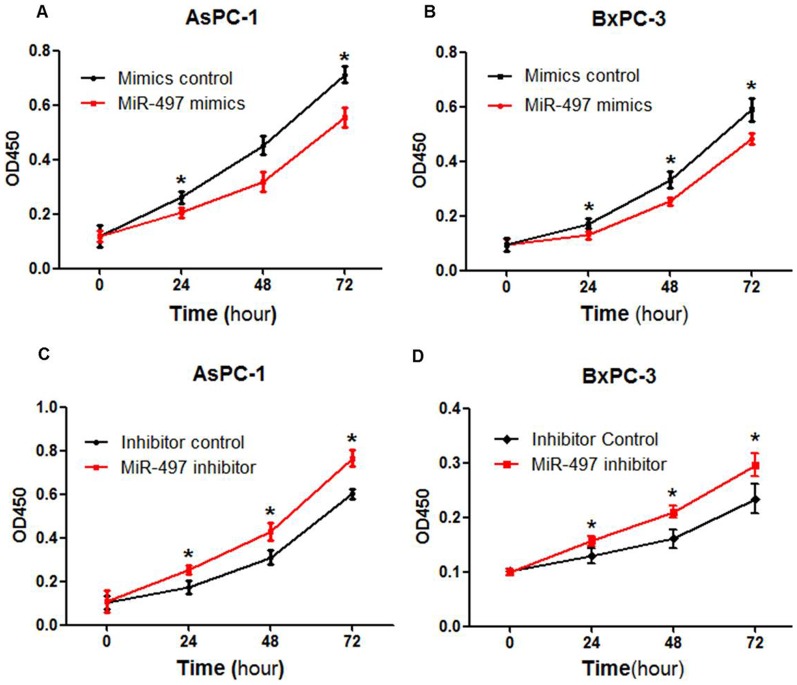
MiR-497 suppressed PDAC cells proliferation. Proliferation was analyzed by CCK-8 assay. (**A, B**) Transfection with miR-497 mimics suppressed PDAC cells proliferation in AsPC-1 and BxPC-3 cells, respectively. (**C, D**) Transfection with miR-497 inhibitor promoted PDAC cells proliferation. (* *P*<*0.05*).

After 48 hours of transfection with miR-497 mimics or inhibitor, miR-497 levels were significantly upregulated or downregulated (**Figure S1 A, B**). Furthermore, miR-497 upregulation significantly inhibited proliferation of PDAC cells (**Figure 2 A, B**). In contrast, miR-497 downregulation promoted the proliferation (**Figure 2 C, D**).

### MiR-497 promoted sensitivity to gemcitabine

**Figure 3:**
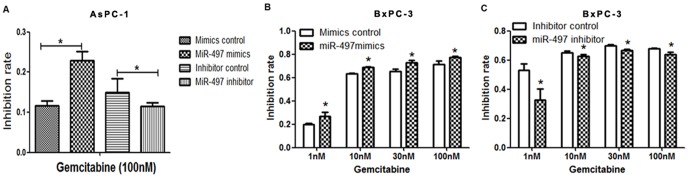
MiR-497 promoted chemosensitivity of gemcitabine. (A) AsPC-1 cells were transfected for 24 hours, and then treated with 100nM gemcitabine for 48 hours. Upregulation of miR-497 by transfection of mimics re-sensitized cells to gemcitabine. Downregulation of miR-497 by transfection of inhibitor decreased the sensitivity of cells to gemcitabine. (**B**) BxPC-3 cells were transfected for 24 hours, and treated with serially diluted gemcitabine (1nM, 10nM, 30nM, 100nM). Cells transfected with miR-497 mimics were more sensitive to gemcitabine. (**C**) Cells transfected with miR-497 inhibitor were more resistant to gemcitabine. (* *P*<*0.05*).

After gemcitabine treatment for 48 hours, the inhibition rates of PDAC cells transfected with miR-497 mimics were significantly higher than cells transfected with mimics control **(** **Figure 3 A, B** **)**. Cells transfected with miR-497 inhibitor were more resistant to gemcitabine treatment than cells transfected with inhibitor control. (**Figure 3 A, C**).

### MiR-497 increased expression of cleaved caspase-3 and PARP activated by gemcitabine

**Figure 4:**
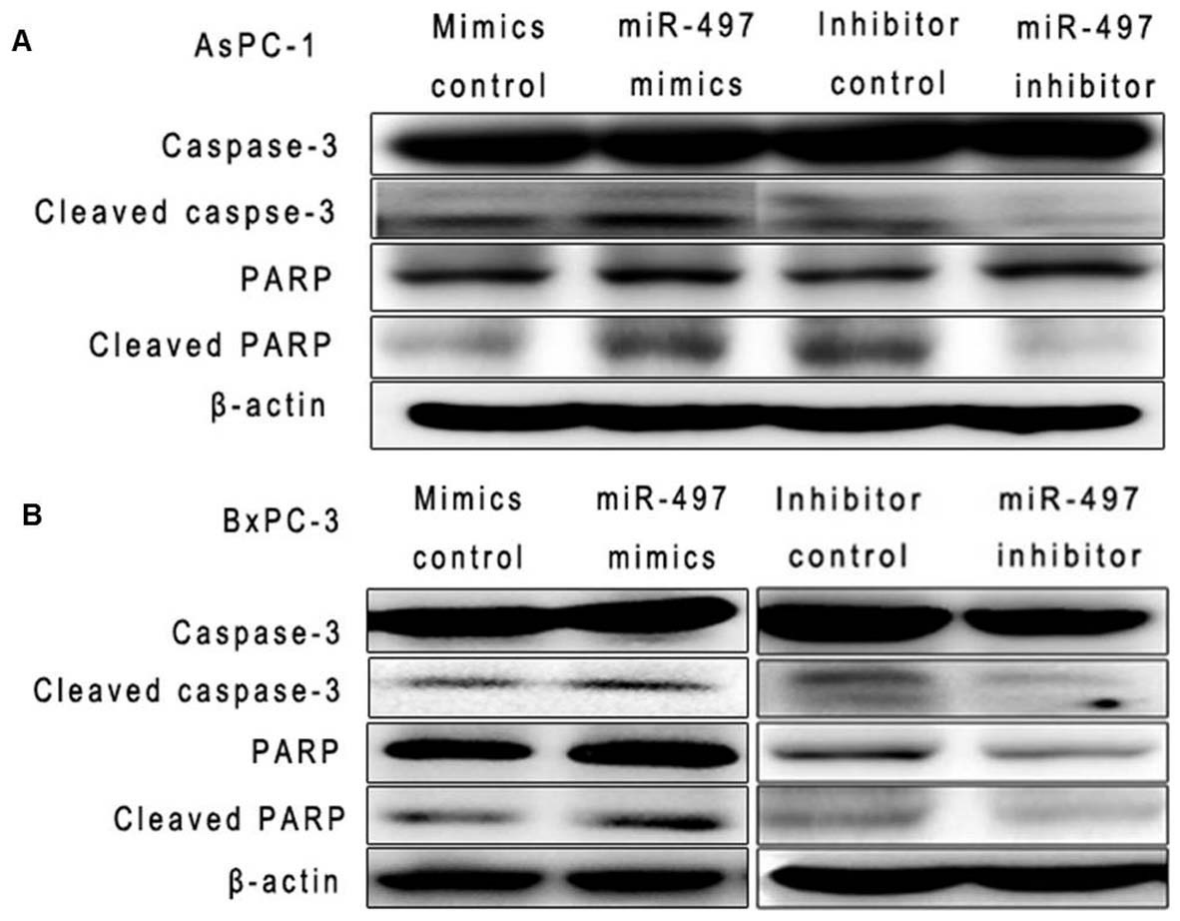
MiR-497 increased the levels of cleaved caspase-3 and PARP. Cells were transfected into 6-well plates. At 24 hours after transfection, AsPC-1 and BxPC-3 cells were treated with 10 μM and 10 nM gemcitabine, respectively. After additional 48 hours, cells were harvested and total protein was extracted. (**A**) Upregulation of miR-497 by transfection of mimics in AsPC-1 cells increased expression of cleaved caspase-3 and PARP, whereas inhibition of miR-497 by transfection of inhibitor decreased expression of cleaved caspase-3 and PARP**,** without any effect on the levels of total caspase-3 and PARP. (**B**) Same results were observed in BxPC-3 cells.

Caspase-3 and Poly (ADP-ribose) polymerase (PARP) are essential for apoptosis. Transfected cells were treated with gemcitabine for 48 hours, and then the levels of cleaved caspase-3 and PARP were detected by western blotting. We found that upregulation of miR-497 increased the levels of cleaved caspase-3 and cleaved PARP (**Figure 4 A, B** **, Figure S2**). Conversely, downregulation of miR-497 decreased the levels of cleaved caspase-3 and cleaved PARP. (**Figure 4 A, B** **, Figure S2**).

### MiR-497 suppressed IGF-1R protein expression by binding to the 3′-UTR

**Figure 5:**
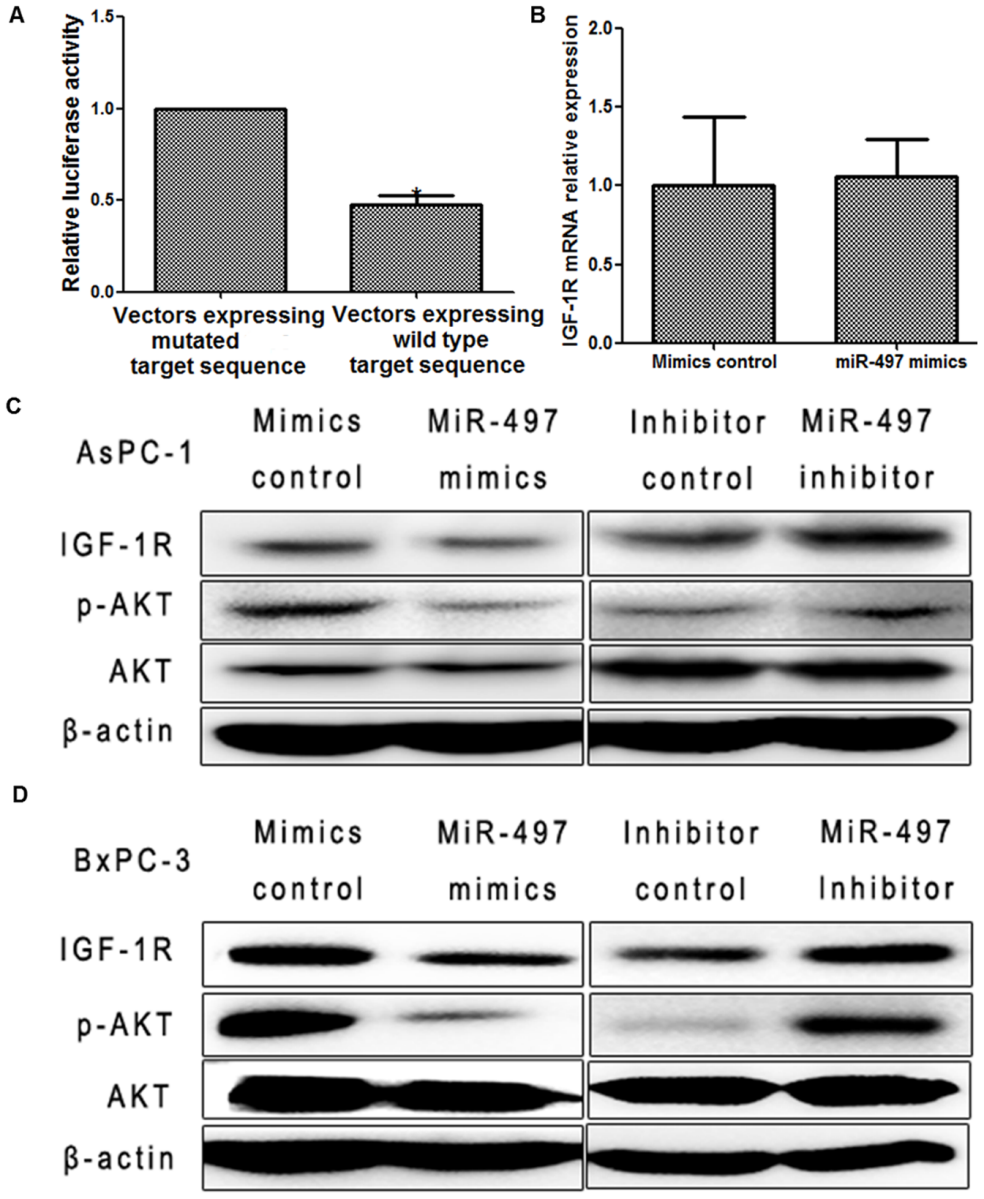
MiR-497 suppressed IGF-1R/AKT pathway. (**A**) Relative luciferase activity was significantly decreased after co-transfection of miR-497 mimics and vectors expressing wild type target sequence in BxPC-3 cells, compared with that in cells co-transfected with mimics and vectors expressing mutated target sequence. (* *P*<*0.05*). (**B**) The mRNA levels of IGF-1R were detected by qRT-PCR. GAPDH was served as an internal control. Transfection with mimics in BxPC-3 cells did not suppress the expression of IGF-1R mRNA. (**C**) The expression levels of IGF-1R, p-AKT and AKT were detected. β-actin was used as an internal control. Upregulation of miR-497 by transfection of mimics in AsPC-1cells significantly inhibited the levels of IGF-1R and p-AKT, whereas downregulation of miR-497 by transfection of inhibitor increased the levels of IGF-1R and p-AKT, without any effect on the levels of total AKT. (**D**) Same results were displayed in BxPC-3 cells.

According to the prediction of open access databases (TargetScan, miRBase Targets, PicTarget, microRNA.org), IGF-1R was considered as a candidate target of miR-497. To confirm this prediction, we performed a luciferase reporter assay. We found luciferase activity was significantly decreased after co-transfection of miR-497 mimics and vectors expressing wild type target sequence in BxPC-3 cells, compared with that in cells co-transfected with mimics and vectors expressing mutated target sequence (*P<0.05*) (**Figure** **5A**). These findings indicated that IGF-1R was a direct target of miR-497.

To further confirm, western blotting was performed. We found that upregulation of miR-497 suppressed the expression of IGF-1R protein without any change in the expression of IGF-1R mRNA (**Figure** **5 B, C, D** **, Figure S3**). In contrast, downregulation of miR-497 increased IGF-1R protein expression (**Figure** **5 C, D** **, Figure S3**). Additionally, we investigated the level of p-AKT mediated by IGF-1R. We observed that the level of p-AKT decreased significantly after upregulation of miR-497. On the contrary, inhibition of miR-497 increased the level of p-AKT (*P<0.05*) (**Figure** **5 C, D** **, Figure S3**).

### The plasma IGF-1R levels in patients with pancreatic cancer

**Figure 6:**
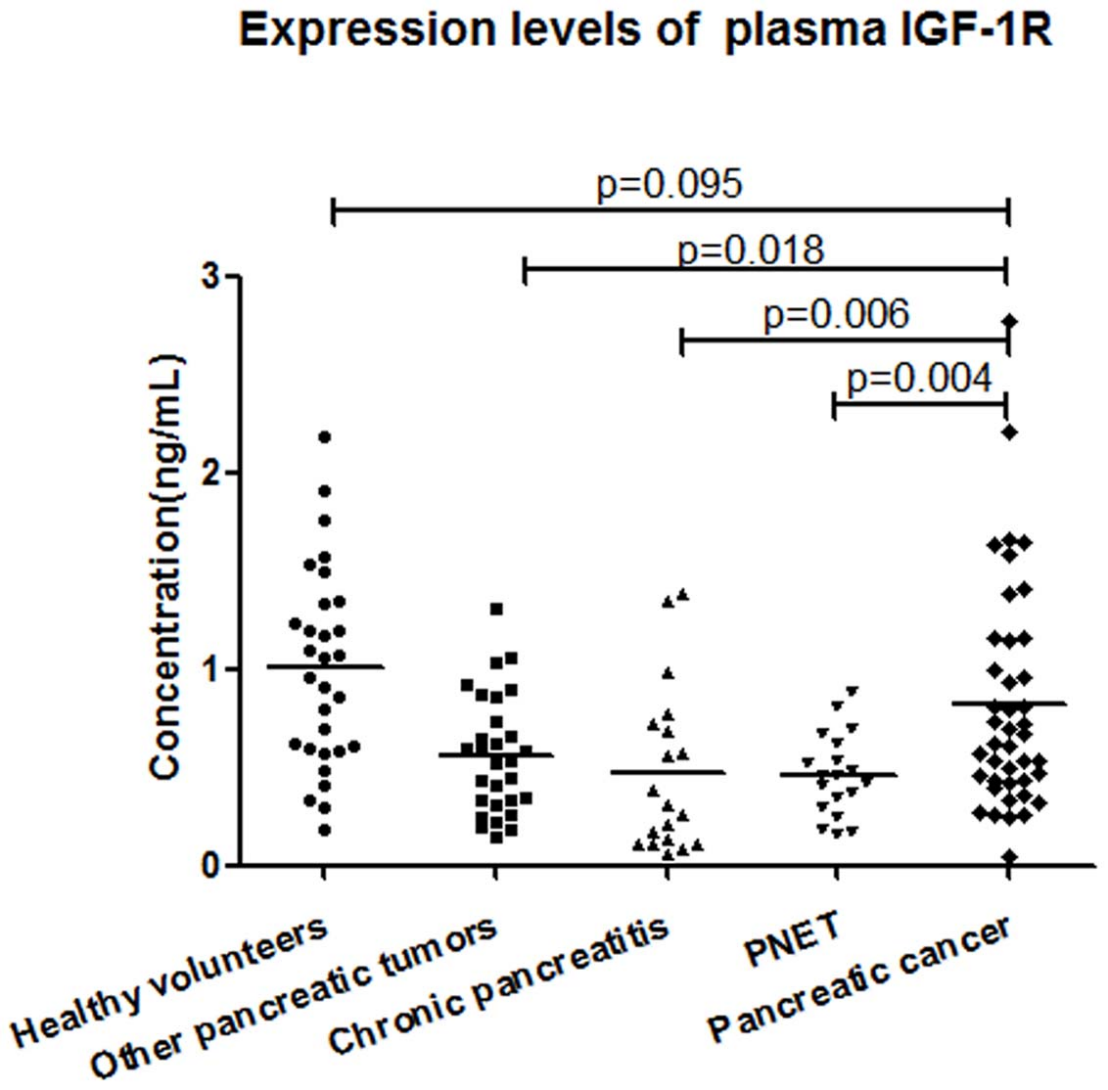
The plasma levels of IGF-1R. Plasma IGF-1R levels were detected by ELISA. The levels of plasma IGF-1R in pancreatic cancer patients were comparable to that in healthy volunteers, but higher than that in patients with chronic pancreatitis, other pancreatic tumors and PNET. Other pancreatic tumors included serous cystadenoma, mucinous cystadenoma, solid pseudopapillary tumor and intra-ductal papillary mucinous neoplasm. PNET, pancreatic neuroendocrine tumors.

Plasma IGF-1R levels were detected by ELISA. The plasma levels of IGF-1R in patients with pancreatic cancer, chronic pancreatitis, other pancreatic tumors, PNET and healthy volunteers were 0.823±0.57 ng/ml, 0.472±0.42 ng/ml, 0.562±0.3 ng/ml, 0.460±0.21 ng/ml, 1.004±0.50 ng/ml, respectively. The levels in patients with pancreatic cancer increased significantly, compared with that in patients with chronic pancreatitis, other pancreatic tumors and PNET (*P = 0.006*, *P = 0.018*, and *P =  0.004*, respectively.). There was no significant difference of plasma IGF-1R levels between patients with pancreatic cancer and healthy volunteers (*P = 0.095*). (**Figure 6** **).**

### The diagnostic value of plasma IGF-1R

The diagnostic value of plasma IGF-1R was evaluated using ROC curve. We showed that plasma IGF-1R displayed a value for distinguishing pancreatic cancer from chronic pancreatitis and PNET (AUC = 0.713, 95% CI: 0.564–0.862, *P = 0.008*; AUC = 0.711, 95% CI: 0.581–0.840, *P = 0.009*, respectively). When cut-off value defined at 0.257 ng/ml, the sensitivity and specificity for distinguishing pancreatic cancer from chronic pancreatitis were 95.2% and 47.3%. When cut-off value defined at 0.699 ng/ml, the sensitivity and specificity for distinguishing pancreatic cancer from PNET were 47.6% and 89.5%. Plasma IGF-1R might also be used in differentiating pancreatic cancer from other pancreatic tumors (AUC = 0.634, 95%CI: 0.504–0.763, *P = 0.057*). When cut-off value defined at 0.925 ng/ml, the sensitivity and specificity for differentiating pancreatic cancer from pancreatic benign tumors were 33.3% and 89.7%.

### The correlation between plasma IGF-1R levels and clinicopathological parameters and survival analysis

Patients were divided into high and low expression groups using the 75^th^ percentile of plasma IGF-1R levels. Tumor locations and TNM stage were associated with plasma IGF-1R levels (*P = 0.013*, *P = 0.01*, respectively. **Table 1**). The median survival time was 18 months, and the 1-, 3-years survival rates were 64% and 23.1%, respectively. The median survival times in high and low expression groups were 11 and 18 months, respectively. No significant difference of overall survival time between high and low expression groups was observed (*P = 0.366*).

**Table 1:** The correlation between plasma IGF-1R levels and clinicopathological parameters.

Variables	Plasma expression of IGF-1R		*P* value
	Low group (32)	High group (10)	
**Sex**			**0.083**
Male	17	2	
Female	15	8	
**Age(years old)**			**0.142**
<65	22	4	
≥65	10	6	
**Locations**			**0.013**
Head	14	9	
Body-tail	18	1	
**Tumor staging**			**0.245**
T1/T2	10	1	
T3/T4	22	9	
**Lymph node staging**			**0.125**
N0	13	1	
N1	19	9	
**M staging**			**1.000**
M0	25	8	
M1	7	2	
**TNM staging**			**0.01**
I/II	22	2	
III/IV	10	8	

## Discussion

Decreased miR-497 expression has been demonstrated in breast, colorectal and cervical cancers [7], [8], [9]. However, there is no report about miR-497 expression levels in pancreatic cancer tissues. Upregulation of miR-497 can suppress cancer cells proliferation, promote apoptosis, decrease migration and invasion capacities and increase chemosensitivity [16], [17], [9]. However, the roles and mechanisms of miR-497 in pancreatic cancer remain unknown. In the current study, we showed miR-497 was significantly downregulated in pancreatic cancer tissues. Upregulation of miR-497 inhibited cells proliferation, enhanced apoptosis and promoted sensitivity to gemcitabine by directly downregulating IGF-1R protein expression.

Our study identified the role of miR-497 in regulating the malignancy of pancreatic cancer. The results supported that miR-497 inhibited cancer cells proliferation, promoted apoptosis and increased chemosensitivity, as reported by literature [9], [16], [17].

Then, we investigated the mechanisms of miR-497 in regulating the progression of pancreatic cancer. We found IGF-1R was a direct target of miR-497 by a luciferase reporter assay, which was also reported in colorectal cancers and cervical cancers [8], [9]. We also performed western blotting for further confirmation. We showed that upregulation of miR-497 decreased IGF-1R protein levels without any change in mRNA expression. Downregulation of miR-497 increased IGF-1R protein levels. Additionally, we investigated the expression of IGF-1R-mediated downstream molecular. We found the level of p-AKT decreased significantly after upregulation of miR-497. Inhibition of miR-497 increased the expression of p-AKT. Therefore, we speculated that miR-497 attenuated the malignancy of pancreatic cancer by partly suppressing IGF-1R/AKT pathway.

IGF-1R/AKT pathway has been identified to involve in the regulation of multiple biological processes of cancers [18], [19]. AKT can activate BAD by phosphorylation on Ser136 [20] or activate NF-κB via regulating IκB kinase (IKK) [21], thus results in anti-apoptotic effects. AKT pathway also involves in chemoresistance. AKT2 inhibition abrogates gemcitabine-induced activation of AKT2 and NF-κB, and enhances gemcitabine-induced PUMA (p53-upregulated modulator of apoptosis) upregulation, resulting in chemosensitization of pancreatic cancers to gemcitabine [22]. Consistent with these studies, our data indicated that the inhibition of IGF-1R/AKT pathway might partly account for the effects of miR-497 on pancreatic cancer cells. However, miR-497 can also attenuate the malignancy of cancers by regulating other targets, such as TARBP2, DICER, BCL2, CCND1, CCNE1, CDC25A, CCND3, CDK4. [23], [24], [25].

Increased IGF-1R expression has been found in many cancers [26] and displays prognostic values [27]. High expression of IGF-1R in human PDAC tissues has also been reported [11] and associates with higher tumor grade and poor survival [12]. However, plasma IGF-1R levels in pancreatic cancer patients have not been detected. Our study showed that plasma IGF-1R levels in patients with pancreatic cancer increased significantly, compared with that in patients with chronic pancreatitis, other pancreatic tumors and PNET. But there was no significant difference of plasma IGF-1R levels between pancreatic cancer patients and healthy volunteers, which might be elucidated partly by that IGF-1R was generally expressed in normal tissues, such as liver, endometrium and neural cells [28]. The value of plasma IGF-1R at diagnosis of pancreatic cancer was evaluated in the current study. There was a potential value for distinguishing pancreatic cancer from chronic pancreatitis, PNET and other pancreatic tumors. But the diagnostic sensitivity or specificity was not perfect, large sample detections were needed to further identify the diagnostic value of plasma IGF-1R.

TNM stage was associated with plasma IGF-1R levels in our study. Patients with advanced stage tumors had high levels of plasma IGF-1R. Plasma IGF-1R could be a new biomarker for guiding TNM stage of pancreatic cancer. Surprisingly, high expression of plasma IGF-1R was not an adverse prognostic factor in the present study.

## Conclusion

MiR-497 was significantly downregulated in pancreatic cancer tissues. Upregulation of miR-497 suppressed the malignancy of pancreatic cancer and re-sensitized PDAC cells to gemcitabine by directly downregulating IGF-1R protein expression. Plasma IGF-1R levels in pancreatic cancer patients increased, and displayed potential values for distinguishing pancreatic lesions. IGF-1R could be also a new plasma biomarker for guiding TNM stage of pancreatic cancer.

## Supporting Information

Figure S1 **The expression level of miR-497 after transfection of mimics or inhibitor.** MiR-497 expression was detected by qRT-PCR. U6 was served as an internal control. (**A**) BxPC-3 cells transfected with miR-497 mimics showed an increase in miR-497 expression. (**B**) Cells transfected with miR-497 inhibitor showed a decrease in miR-497 expression. Data were shown as mean±SD. (* *P<0.05*).
(TIF)

Figure S2 **Relative expression levels of cleaved caspase-3 and PARP.** Data were shown as mean±SD. (**A**) The relative expression levels of proteins in AsPC-1 cells. (**B**) The relative expression levels of proteins in BxPC-3 cells. (* *P<0.05*).
(TIF)

Figure S3 **Relative expression levels of IGF-1R and p-AKT.** Relative expression levels were shown as mean±SD. (**A**) The relative levels of IGF-1R and p-AKT in AsPC-1 cells. (**B**) The relative levels of proteins in BxPC-3 cells. (* *P<0.05*).
(TIF)

## References

[1] Trajkovic-Arsic M, Kalideris E, Siveke JT (2013) The role of insulin and IGF system in pancreatic cancer. J Mol Endocrinol 50: R67–74. 23493758 10.1530/JME-12-0259

[2] Costello E, Greenhalf W, Neoptolemos JP (2012) New biomarkers and targets in pancreatic cancer and their application to treatment. Nat Rev Gastroenterol Hepatol 9: 435–444. 22733351 10.1038/nrgastro.2012.119

[3] Winter JM, Yeo CJ, Brody JR (2013) Diagnostic, prognostic, and predictive biomarkers in pancreatic cancer. J Surg Oncol 107: 15–22. 22729569 10.1002/jso.23192

[4] Macgregor-Das AM, Iacobuzio-Donahue CA (2013) Molecular pathways in pancreatic carcinogenesis. J Surg Oncol 107: 8–14. 22806689 10.1002/jso.23213 PMC3661191

[5] Siegel R, Naishadham D, Jemal A (2013) Cancer statistics, 2013. CA Cancer J Clin 63: 11–30. 23335087 10.3322/caac.21166

[6] Iorio MV, Croce CM (2012) MicroRNA dysregulation in cancer: diagnostics, monitoring and therapeutics. A comprehensive review. EMBO Mol Med 4: 143–159. 22351564 10.1002/emmm.201100209 PMC3376845

[7] Li D, Zhao Y, Liu C, Chen X, Qi Y, et al (2011) Analysis of MiR-195 and MiR-497 expression, regulation and role in breast cancer. Clin Cancer Res 17: 1722–1730. 21350001 10.1158/1078-0432.CCR-10-1800

[8] Luo M, Shen D, Zhou X, Chen X, Wang W (2013) MicroRNA-497 is a potential prognostic marker in human cervical cancer and functions as a tumor suppressor by targeting the insulin-like growth factor 1receptor. LID - S0039-6060(12)00751-9 [pii]LID - 10.1016/j.surg.2012.12.004 [doi]. Surgery. 10.1016/j.surg.2012.12.004 23453369

[9] Guo ST, Jiang CC, Wang GP, Li YP, Wang CY, et al (2013) MicroRNA-497 targets insulin-like growth factor 1 receptor and has a tumour suppressive role in human colorectal cancer. Oncogene 32: 1910–1920. 22710713 10.1038/onc.2012.214 PMC3630484

[10] Sachdev D, Yee D (2007) Disrupting insulin-like growth factor signaling as a potential cancer therapy. Mol Cancer Ther 6: 1–12. 17237261 10.1158/1535-7163.MCT-06-0080

[11] Hakam A, Fang Q, Karl R, Coppola D (2003) Coexpression of IGF-1R and c-Src proteins in human pancreatic ductal adenocarcinoma. Dig Dis Sci 48: 1972–1978. 14627343 10.1023/a:1026122421369

[12] Valsecchi ME, McDonald M, Brody JR, Hyslop T, Freydin B, et al (2012) Epidermal growth factor receptor and insulin like growth factor 1 receptor expression predict poor survival in pancreatic ductal adenocarcinoma. Cancer 118: 3484–3493. 22086503 10.1002/cncr.26661

[13] Tan MH, Nowak NJ, Loor R, Ochi H, Sandberg AA, et al (1986) Characterization of a new primary human pancreatic tumor line. Cancer Invest 4: 15–23. 3754176 10.3109/07357908609039823

[14] Kleeff J, Wildi S, Friess H, Korc M (1999) Ligand induced upregulation of the type II transforming growth factor (TGF-beta) receptor enhances TGF-beta responsiveness in COLO-357 cells. Pancreas 18: 364–370. 10231841 10.1097/00006676-199905000-00006

[15] Chen WH, Horoszewicz JS, Leong SS, Shimano T, Penetrante R, et al (1982) Human pancreatic adenocarcinoma: in vitro and in vivo morphology of a new tumor line established from ascites. In Vitro 18: 24–34. 7182348 10.1007/BF02796382

[16] Zhao WY, Wang Y, An ZJ, Shi CG, Zhu GA, et al.. (2013) Downregulation of miR-497 promotes tumor growth and angiogenesis by targeting HDGF in non-small cell lung cancer. LID - S0006-291X(13)00780-8 [pii]LID - 10.1016/j.bbrc.2013.05.010 [doi]. Biochem Biophys Res Commun. 10.1016/j.bbrc.2013.05.010 23673296

[17] Shen L, Li J, Xu L, Ma J, Li H, et al (2012) miR-497 induces apoptosis of breast cancer cells by targeting Bcl-w. Exp Ther Med 3: 475–480. 22969914 10.3892/etm.2011.428 PMC3438749

[18] Pollak M (2012) The insulin and insulin-like growth factor receptor family in neoplasia: an update. Nat Rev Cancer 12: 159–169. 22337149 10.1038/nrc3215

[19] Tian X, Hao K, Qin C, Xie K, Xie X, et al (2013) Insulin-Like Growth Factor 1 Receptor Promotes the Growth and Chemoresistance of Pancreatic Cancer. Dig Dis Sci 58: 2705–12. 23589145 10.1007/s10620-013-2673-2

[20] Zhang XH, Chen SY, Tang L, Shen YZ, Luo L, et al (2013) Myricetin Induces Apoptosis in Hepg2 Cells through Akt/P70s6k/Bad Signaling and Mitochondrial Apoptotic Pathway. Anticancer Agents Med Chem 13: 1575–81. 23438827 10.2174/1871520613666131125123059

[21] Faissner A, Heck N, Dobbertin A, Garwood J (2006) DSD-1-Proteoglycan/Phosphacan and receptor protein tyrosine phosphatase-beta isoforms during development and regeneration of neural tissues. Adv Exp Med Biol 557: 25–53. 16955703 10.1007/0-387-30128-3_3

[22] Chen D, Niu M, Jiao X, Zhang K, Liang J, et al (2012) Inhibition of AKT2 Enhances Sensitivity to Gemcitabine via Regulating PUMA and NF-kappaB Signaling Pathway in Human Pancreatic Ductal Adenocarcinoma. Int J Mol Sci 13: 1186–1208. 22312312 10.3390/ijms13011186 PMC3269746

[23] Caramuta S, Lee L, Ozata DM, Akcakaya P, Xie H, et al (2013) Clinical and functional impact of TARBP2 over-expression in adrenocortical carcinoma. Endocr Relat Cancer 20: 551–564. 23671264 10.1530/ERC-13-0098 PMC3709642

[24] Zhu W, Zhu D, Lu S, Wang T, Wang J, et al (2012) miR-497 modulates multidrug resistance of human cancer cell lines by targeting BCL2. Med Oncol 29: 384–391. 21258880 10.1007/s12032-010-9797-4

[25] Furuta M, Kozaki K, Tanimoto K, Tanaka S, Arii S, et al (2013) The tumor-suppressive miR-497-195 cluster targets multiple cell-cycle regulators in hepatocellular carcinoma. PLOS ONE 8: e60155. 23544130 10.1371/journal.pone.0060155 PMC3609788

[26] Pollak M (2008) Insulin and insulin-like growth factor signalling in neoplasia. Nat Rev Cancer 8: 915–928. 19029956 10.1038/nrc2536

[27] Ozkan EE (2011) Plasma and tissue insulin-like growth factor-I receptor (IGF-IR) as a prognostic marker for prostate cancer and anti-IGF-IR agents as novel therapeutic strategy for refractory cases: a review. Mol Cell Endocrinol 344: 1–24. 21782884 10.1016/j.mce.2011.07.002

[28] O'Kusky J, Ye P (2012) Neurodevelopmental effects of insulin-like growth factor signaling. Front Neuroendocrinol 33: 230–251. 22710100 10.1016/j.yfrne.2012.06.002 PMC3677055

