# Supplementary figures and images for: Insulin-Like Growth Factor 1 Receptor (IGF-1R) as a Target of MiR-497 and Plasma IGF-1R Levels Associated with TNM Stage of Pancreatic Cancer
Source: PLoS One. 2014 Mar 25;9(3):e92847. doi: 10.1371/journal.pone.0092847 (PMC3965476; doi:10.1371/journal.pone.0092847)

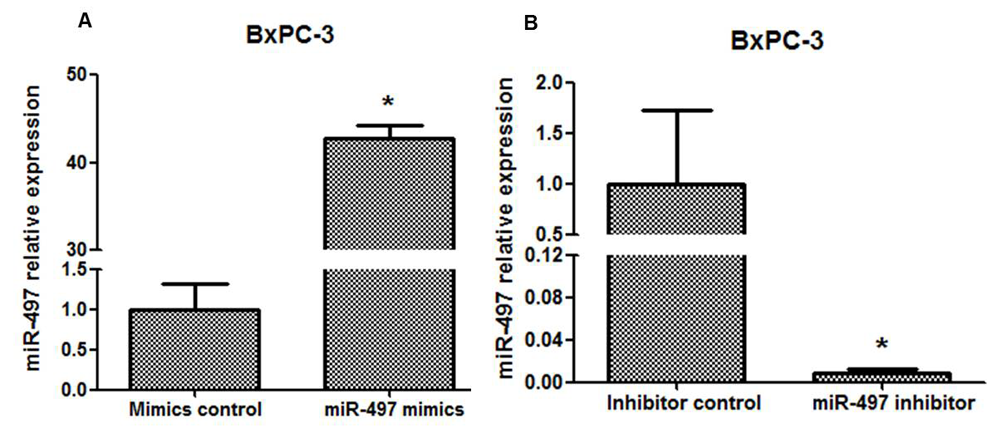

Supplement: Figure S1 — The expression level of miR-497 after transfection of mimics or inhibitor. MiR-497 expression was detected by qRT-PCR. U6 was served as an internal control. (A) BxPC-3 cells transfected with miR-497 mimics showed an increase in miR-497 expression. (B) Cells transfected with miR-497 inhibitor showed a decrease in miR-497 expression. Data were shown as mean±SD. (* P<0.05). (TIF) [file pone.0092847.s001.tif]

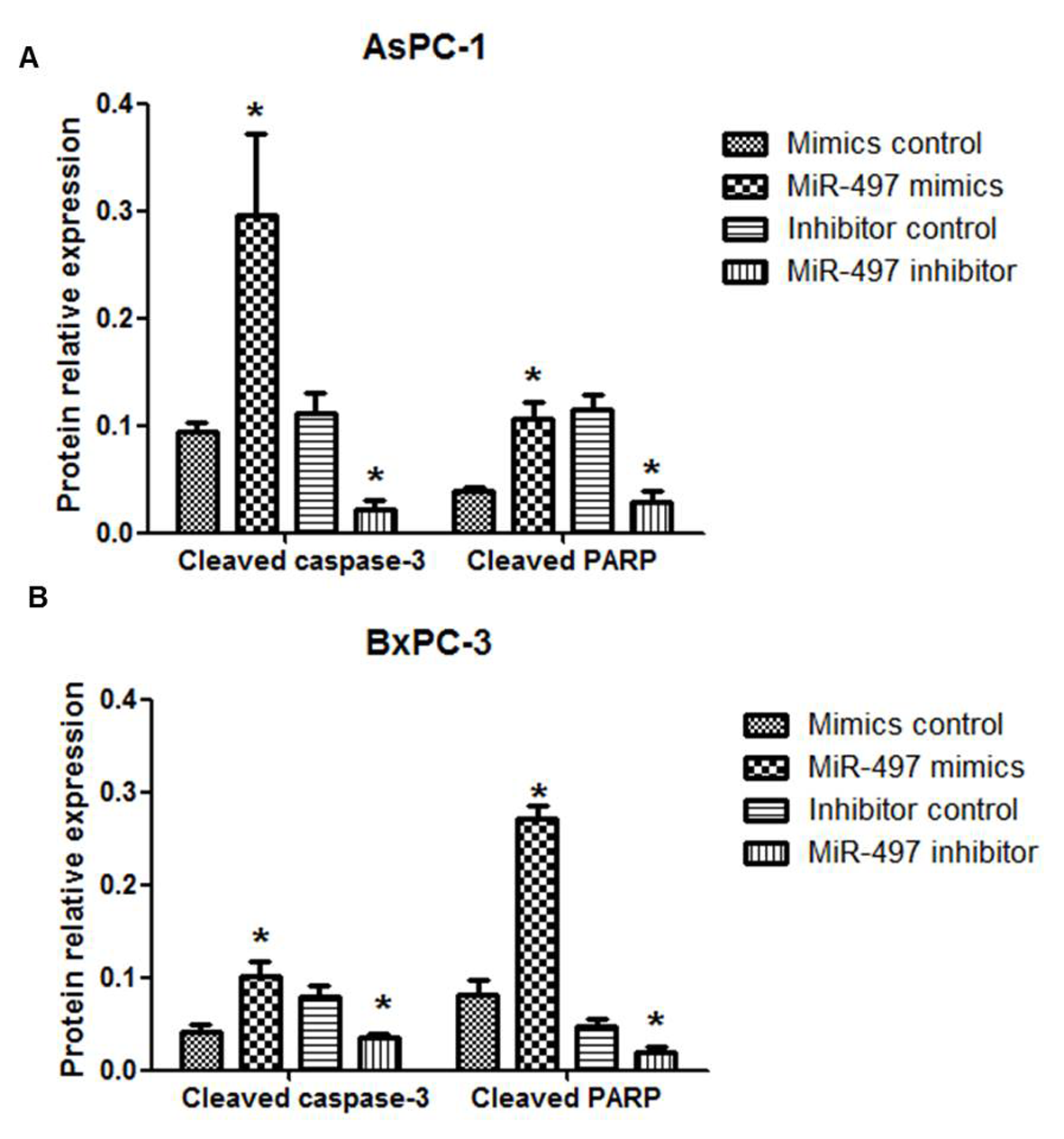

Supplement: Figure S2 — Relative expression levels of cleaved caspase-3 and PARP. Data were shown as mean±SD. (A) The relative expression levels of proteins in AsPC-1 cells. (B) The relative expression levels of proteins in BxPC-3 cells. (* P<0.05). (TIF) [file pone.0092847.s002.tif]

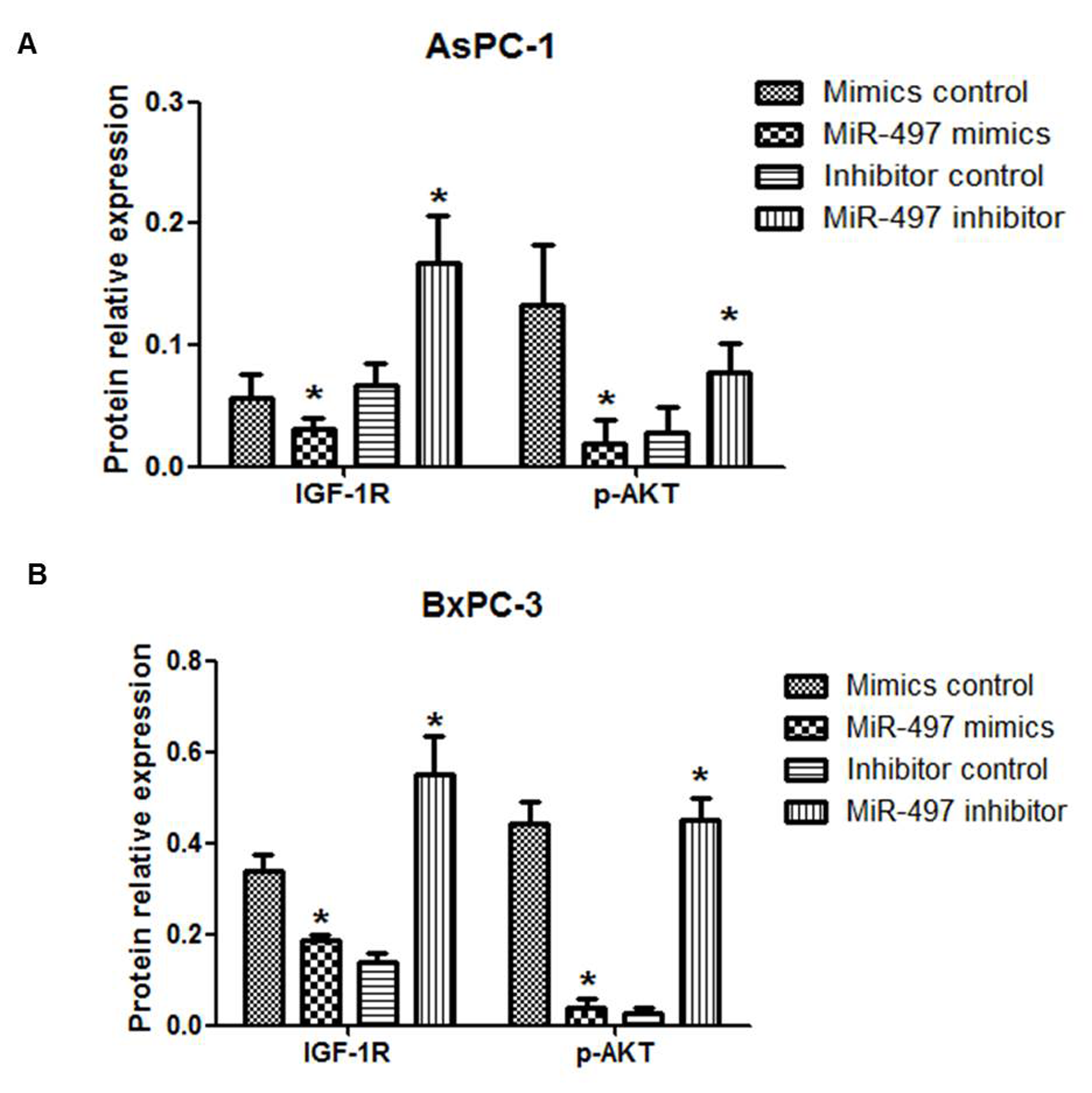

Supplement: Figure S3 — Relative expression levels of IGF-1R and p-AKT. Relative expression levels were shown as mean±SD. (A) The relative levels of IGF-1R and p-AKT in AsPC-1 cells. (B) The relative levels of proteins in BxPC-3 cells. (* P<0.05). (TIF) [file pone.0092847.s003.tif]
